# Feasibility of Glutamate and GABA Detection in Pons and Thalamus at 3T and 7T by Proton Magnetic Resonance Spectroscopy

**DOI:** 10.3389/fnins.2020.559314

**Published:** 2020-10-23

**Authors:** Samaira Younis, Anders Hougaard, Casper E. Christensen, Mark B. Vestergaard, Esben T. Petersen, Vincent O. Boer, Olaf B. Paulson, Messoud Ashina, Anouk Marsman, Henrik B. W. Larsson

**Affiliations:** ^1^Danish Headache Center, Department of Neurology, Rigshospitalet Glostrup, Faculty of Health and Medical Sciences, University of Copenhagen, Glostrup, Denmark; ^2^Functional Imaging Unit, Department of Clinical Physiology, Nuclear Medicine and PET, Rigshospitalet Glostrup, Faculty of Health and Medical Sciences, University of Copenhagen, Glostrup, Denmark; ^3^Danish Research Centre for Magnetic Resonance, Centre for Functional and Diagnostic Imaging and Research, Copenhagen University Hospital Hvidovre, Hvidovre, Denmark; ^4^Center for Magnetic Resonance, Department of Health Technology, Technical University of Denmark, Kongens Lyngby, Denmark; ^5^Neurobiology Research Unit, Department of Neurology, Rigshospitalet, Faculty of Health and Medical Sciences, University of Copenhagen, Copenhagen, Denmark

**Keywords:** brainstem, brain, MRS, Glx, NAA, creatine, choline, myo-inositol

## Abstract

Glutamate detection in pons and thalamus using proton magnetic resonance spectroscopy (^1^H-MRS) after an intervention is of interest for studying various brain disorders. However, ^1^H-MRS in these brain regions is challenging and time-consuming, especially in longitudinal study designs. ^1^H-MRS of more cortical structures at the ultrahigh magnetic field strength of 7T yields an improved spectral output, including separation of the glutamate signal from the glutamine signal, in a shorter and more feasible scan time, as compared to conventional clinical field strengths. For this purpose, we compared the feasibility of ^1^H-MRS at 3T and 7T in pons and thalamus by applying a longitudinal study design of repeated measures on same day and three separate days at both field strength in five healthy participants. Total ^1^H-MRS acquisition time was reduced by a factor 3.75 for pons and by a factor 3 for thalamus at 7T as compared to 3T. We found higher spectral signal-to-noise ratio (SNR) (*p* < 0.001), lower linewidth (*p* = 0.001) and lower Cramér–Rao lower bounds (CRLB) (*p* < 0.001) for the combined glutamate and glutamine signal (Glx) in thalamus at 7T as compared to 3T. In pons, CRLB of Glx and SNR were lower at 7T (*p* = 0.002 and *p* = 0.006), with no differences in linewidth compared to 3T. Mean within-subject variability of Glx concentration estimates was lower at 7T compared to 3T for both pons and thalamus. At 7T, it was possible to assess glutamate and γ-aminobutyric acid (GABA) simultaneously in pons and thalamus. In conclusion, ^1^H-MRS at 7T resulted in improved spectral quality while allowing shorter scan times than at 3T as well as estimation of the pure glutamate signal in pons and thalamus. This opens up the opportunity for multimodal study designs and multiregional subcortical ^1^H-MRS research. Glutamate and GABA measurement at 7T in pons and thalamus is advantageous for future investigations of excitatory–inhibitory mechanisms in brain disorders.

## Introduction

Non-invasive, *in vivo* measurement of glutamate, the major excitatory neurotransmitter, using proton magnetic resonance spectroscopy (^1^H-MRS) is of interest for studying the mechanisms behind various brain disorders as well as the pharmacokinetics of drugs acting within the central nervous system (Foerster et al., 2013; Brennan et al., 2017; Prisciandaro et al., 2017; Younis et al., 2018). Glutamatergic changes in subcortical structures such as pons and thalamus are particularly of interest (Pioro et al., 1999; Foerster et al., 2013; Adanyeguh et al., 2015; Neill et al., 2016; Bathel et al., 2018; Younis et al., 2018). However, current literature is lacking knowledge about the feasibility of longitudinal pharmacokinetic studies using ^1^H-MRS in pons and thalamus. As such, the potential of using ultrahigh field 7T magnetic resonance imaging (MRI) scanners to study pontine and thalamic glutamatergic changes by ^1^H-MRS is also unknown.

Acquiring ^1^H-MRS data of sufficient quality from subcortical areas demands longer scan durations, compared to cortical structures, as ^1^H-MRS of deep brain regions is technically more challenging. These include increased magnetic field inhomogeneity and increased distance from the head coils and, as such, decreased coil sensitivity. In addition to the technical challenges of deep brain regions, the longitudinal design of pharmacokinetic ^1^H-MRS studies further adds to the total scan time. Ultrahigh field ^1^H-MRS will likely improve the spectral quality, while requiring shorter scan times, compared to clinical field strengths, such as 1.5T and 3T (Mekle et al., 2009; Pradhan et al., 2015; Terpstra et al., 2016). This may open up the opportunity to study pharmacokinetic effects in multiple brain regions and/or include other modalities that complement the investigations in multimodal study designs (Mangia et al., 2009; Cleve et al., 2017).

Additionally, measurement of the brain glutamate signal is arduous due to its overlap with the glutamine signal at clinical field strengths, where the combined signal (Glx) is commonly reported instead (Tkác et al., 2001). While Glx mainly represents the glutamate signal (∼80%) (Danbolt, 2001), the separation of glutamate from glutamine is needed for proper characterization of potential excitatory abnormalities in neurological disorders (Merritt et al., 2016; Zielman et al., 2017; Mahone et al., 2018) due to involvement of glutamine in the glutamate metabolism (Zhou and Danbolt, 2014).

The improved spectral quality at 7T allows for separation of the glutamate signal from the glutamine signal (Tkác et al., 2001, Tkáč et al., 2009; Snyder and Wilman, 2010; Deelchand et al., 2014). While this has been demonstrated in studies comparing cortical 3T ^1^H-MRS to 7T ^1^H-MRS, and by using shorter scan times at 7T compared to 3T (Mekle et al., 2009; Pradhan et al., 2015; Terpstra et al., 2016), the findings are not readily applicable to pons and thalamus due to increased susceptibility effects at higher field strengths in subcortical regions (Sclocco et al., 2018). Moreover, estimation of inhibitory neurotransmitter γ-aminobutyric acid (GABA) levels using non-edited ^1^H-MRS is potentially a possibility at 7T in subcortical areas (van de Bank et al., 2015). This is particularly useful for pharmacological studies investigating the relationship between excitatory and inhibitory mechanisms.

In the present study, we applied a longitudinal study design with repeated measurements (Younis et al., 2018). We applied ^1^H-MRS sequences tailored for pontine and thalamic investigations at 3 and 7T, which is pertinent in a clinical setting. Spectral quality was expected to remain similar or improve in pons and thalamus while using shorter scan times at 7T compared to 3T. We investigated whether pontine and thalamic glutamate and GABA could be assessed at 7T using non-edited ^1^H-MRS, and estimated the robustness of the measurements using scan times that were a factor 3.75 (pons) and a factor 3 (thalamus) shorter at 7T compared to 3T. Based on the variability estimates from the study it will be possible to calculate detection thresholds of pontine and thalamic levels of glutamate and GABA in future longitudinal study designs at 7T.

## Materials and Methods

### Participants

Healthy participants were recruited from a Danish website designed for recruitment of participants to health research^1^ and were offered participation in the 7T study after completion of the 3T study investigating drug-induced metabolic changes in pons and thalamus (Younis et al., 2018). Five participants continued to the 7T study (3 women, median age = 26 years, age range = 21–30 years). Inclusion criteria were age between 18 and 50 years and weight between 50 and 100 kg. Exclusion criteria were primary headache disorders according to the diagnostic criteria of the beta version of the third *International Classification of Headache Disorders* (*ICHD-3* beta) (Headache Classification Committee of the International Headache Society (IHS), 2013) (except episodic tension-type headache < 2 days per month during the last year), first-degree family members with primary headache disorders according to *ICHD-3* beta (except episodic tension-type headache for < 6 days per month), daily intake of medication (except contraceptives), no contraception use, history of cardiovascular, cerebrovascular or psychiatric disease, drug abuse, and MRI contraindications (including braces and teeth implants, which could cause magnetic field inhomogeneities in the pons).

### Ethical Approval

The study was approved by the Danish National Committee on Health Research Ethics (H-15019063) and the Danish Medicines Agency (CIV-16-12-017964). The study is registered at www.clinicaltrials.gov (NCT03143465) and is part of a larger study where other parts have been and/or will be published elsewhere. All participants provided written consent after receiving oral and written information in accordance with the Declaration of Helsinki of 1964 with subsequent revisions.

### Experimental Design

Participants underwent the longitudinal study design at both 3T and 7T yielding a total of 6 study days with three MRI sessions on each study day. Study days 1–3 were consecutively completed at 3T and study days 4–6 were consecutively completed at 7T. The 7T study was initiated 10 months after completion of the 3T study. As the study was part of a larger, predefined setup (see section “Ethical Approval”), which included an MR angiography, a double-blind, placebo-controlled, randomized, double-dummy, three-way cross-over design was applied including calcitonin gene-related peptide, sildenafil and placebo completed on three separate days. On each study day, participants were scanned once before (baseline) and twice (scan 1 and scan 2), at fixed time points, after drug administration (40 and 140 min) yielding a total of three MRI sessions per study day. Hence, the complete study setup was performed at both field strengths. This included scans (scan 1 and scan 2) after active drugs, which were not included for variability estimates due to possible drug induced changes (Younis et al., 2018). However, the data were pertinent for spectral quality investigations.

Detailed information on the experimental design is published and available (Younis et al., 2018).

### MR Acquisition

3T and 7T whole-body MR scanners were used (Philips Achieva, Best, the Netherlands; and Cleveland, OH, United States, respectively). A 32-channel phase array head coil was used at 3T (Philips, Best, Netherlands) and a two-channel volume transmit head coil with 32-channel receiver array was used at 7T (Nova Medical, Inc., Burlington, MA, United States).

### Structural Imaging

3D T_1_-weighted magnetization prepared turbo field echo sequences were acquired at 3T and 7T before ^1^H-MRS for voxel placement (3T: field of view (FOV) = 240 × 240 × 170 mm^3^, voxel size = 1.00 × 1.08 × 1.10 mm^3^, TR = 8.0 ms, TE = 3.7 ms, flip angle = 8°; 7T: FOV = 256 × 256 × 190 mm^3^, voxel size = 1.0 × 1.0 × 1.0 mm^3^, TR = 8.0 ms, TE = 3.03 ms, flip angle = 7°). The structural images were used for MRS voxel placement. To ensure that voxels were placed in the same area screenshots were saved for all scans, both within and between subjects as well as between field strengths, and voxels were placed by the same investigator.

### ^1^H-MRS

A point-resolved spectroscopy (PRESS) pulse sequence was used at 3T, as previously reported (Younis et al., 2018; Table 1). In pons, the following sequence parameters were used: voxel size = 10.5 × 12.5 × 22 mm^3^, TR = 3,000 ms, TE = 37 ms, 480 acquisitions, scan duration = 24 min. In thalamus the sequence parameters were: voxel size = 16 × 12 × 16 mm^3^, TR = 3,000 ms, TE = 35 ms, 192 acquisitions, scan duration = 9 min 36 s. First-order shimming was performed on the ^1^H-MRS voxels. Water suppression using automated water suppression optimization prescans added approximately 5 min to the sequence. The unsuppressed water signal was obtained from both ^1^H-MRS voxels and used as internal reference for quantifying the measured metabolites (Christiansen et al., 1993). The protocol was optimized to target small deep brain areas and reduce potential partial volume effects by using relatively small voxels and a relatively large number of acquisitions.

**TABLE 1:** Overview of ^1^H-MRS sequences at 3T and 7T.

	Pons		Thalamus	
	3T	7T	3T	7T
Sequence	PRESS	sLASER	PRESS	sLASER
TR (ms)	3,000	3,600	3,000	3,600
TE (ms)	37	30	35	30
Shimming	First-order	Second-order	First-order	Second-order
Acquisitions	480	128	192	64
Total scan duration (incl. water suppression)	∼29 min^a^	7 min 41 s	∼15 min^a^	3 min 50 s
Voxel size (mm^3^)	10.5 × 12.5 × 22	10.5 × 12.5 × 22	16 × 12 × 16	16 × 12 × 16

*^a^Includes additional ∼5 min due to automatic water suppression optimization. PRESS, point-resolved spectroscopy; sLASER, semilocalization by adiabatic-selective refocusing.*

At 7T, a semilocalization by adiabatic-selective refocusing (sLASER) sequence was used (Boer et al., 2011). For pons, the following sequence parameters were applied: voxel size = 10.5 × 12.5 × 22 mm^3^, TR = 3,600 ms, TE = 30 ms, 128 acquisitions, scan duration = 7 min 41 s. TR was slightly increased compared to 3T due to higher specific absorption rate (SAR) at 7T. For thalamus the following sequence parameters were used: voxel size = 16 × 12 × 16 mm^3^, TR = 3,600 ms, TE = 30 ms, 64 acquisitions, scan duration = 3 min 50 s. A second-order shim was applied. Variable power RF pulses with optimized relaxation delays were used for water suppression without automatic optimization. Unsuppressed water signal was acquired with an additional single acquisition (Tkác et al., 1999), which did not add noticeable time to the total scan duration.

**FIGURE 1:**
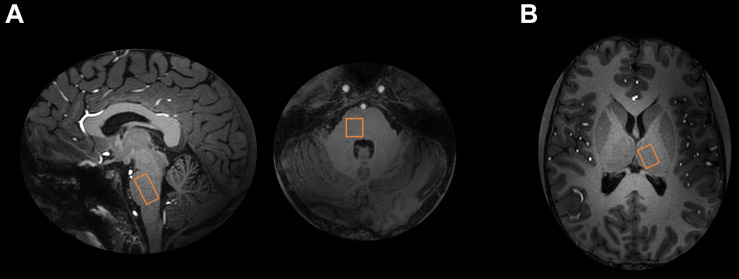
^1^H-MRS voxel locations. **(A)** Pontine and **(B)** thalamic ^1^H-MRS voxels are shown on 7T T1 image.

In pons, the voxel was placed in the right unilateral pons (Figure 1). In thalamus, the voxel was placed in the left, contralateral, side in accordance with the trigeminal pain pathway.

### Spectral Fitting and Quantification

Retrospective artifact detection was performed for 3T and 7T spectra (Tapper et al., 2018). Visual inspection was performed to exclude poor quality spectra. LCModel (Version 6.3-1F) was used for quantification of metabolite levels (Provencher, 2018). Simulated basis sets for both 3T and 7T data included the following metabolites and a measured macromolecular baseline: alanine, aspartate, ascorbate, glycerophosphocholine, phosphocholine, creatine, phosphocreatine, GABA, glutamine (Gln), glutamate (Glu), glycine, glutathione, myo-inositol, lactate, *N-*acetyl aspartate, *N*-acetylaspartyl glutamate, phosphorylethanolamine, scylloinositol, taurine, and serine. The basis sets were simulated with FID-A (Simpson et al., 2017). J-coupling and chemical shift values were derived from Govindaraju et al. (2000). The macromolecular baseline from van de Bank et al. (2015) was used for analysis of the 7T data. The macromolecule quantification implemented in LCModel was applied for analysis of the 3T data (Provencher, 2018). We used realistic pulse shapes and chemical shift displacement effects were considered. The spectra were fitted between 0.2 and 0.4 ppm with a knot spacing of 0.2.

Following metabolites were used for further statistical analyses: Glx (glutamate + glutamine), glutamate, glutamine, GABA, and major spectral components: total creatine (tCreatine; creatine + phosphocreatine), total NAA (tNAA; *N-*acetyl aspartate + *N*-acetylaspartyl glutamate), total choline (tCholine; glycerophosphocholine + phosphocholine), myo-inositol and lactate. Metabolite concentrations, which could not be estimated, were excluded from all further analyses (Provencher, 2018).

**FIGURE 2:**
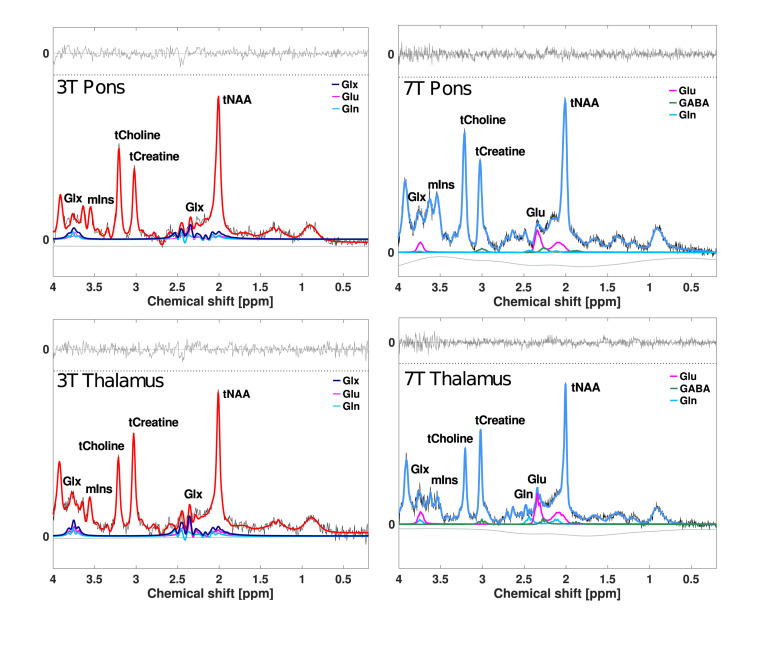
Examples of ^1^H-MR spectra from pons and thalamus. All lines represent the LCModel fits. Red lines represent the overall fit at 3T, and blue lines represent the overall fit at 7T. Purple, pink, light blue, and green lines represent fits for Glx, glutamate, glutamine, and GABA. Baselines are visualized as lower horizontal wavy gray lines in all spectra. Residual fits are presented as upper horizontal gray lines. SNR and FWHM of 3T example spectra are 20 and 0.03 ppm in pons and 17 and 0.03 in thalamus. SNR and FWHM of 7T example spectra are 25 and 0.04 ppm in pons and 25 and 0.03 in thalamus. Glx, glutamate + glutamine; Glu, glutamate; Gln, glutamine; GABA, γ-aminobutyric acid; tNAA, *N*-acetylaspartate + *N*-acetylaspartyl glutamate; tCreatine, creatine + phosphocreatine; tCholine, glycerophosphocholine + phosphocholine; mIns, myo-inositol; SNR, signal-to-noise ratio; FWHM, full-width of half-maximum, linewidth.

Signal-to-noise ratio (SNR), linewidth (full-width of half-maximum; FWHM) and Cramér–Rao lower bounds (CRLB) of all spectra were extracted from LCModel and used to assess spectral quality. Metabolite concentration estimates were excluded from variability analyses when CRLB was 20 or higher (Provencher, 2018). Concentration estimates with CRLB of < 50 were included for the variability analysis of glutamine and GABA due to their relatively low signals (Kreis, 2004; Tkáč et al., 2009; Provencher, 2018). Representative examples of 3T and 7T ^1^H-MR spectra obtained from pons and thalamus are presented in Figure 2.

### Statistical Analyses

Primary end-points were differences in the spectral quality measures (SNR, FWHM, and CRLB) between 3T and 7T in pons and thalamus. A linear mixed model was used for each metabolite with the magnetic field strength as the fixed effect, and subjects and study day nested within subjects as random effects. Secondary end-point was within-subject coefficient of variation (CV) at 3T and 7T for metabolites based on placebo day data (baseline, scan 1 and scan 2) and baseline measurements of CGRP and sildenafil days, thus excluding active drug data.

We further explored the differences in metabolic concentration estimates at 3T and 7T based on placebo day data (baseline, scan 1 and scan 2) and baseline measurements on CGRP and sildenafil days. A linear mixed model was used with magnetic field strength as the fixed effect, and subjects and study day nested within subjects as random effects. The small sample size (*n* = 5) in the present study inhibited us from performing exploratory statistical tests of metabolic changes after drug administration to evaluate the reproducibility of previous findings observed at 3T (Younis et al., 2018) on the 7T data.

R (version 3.5.1) was used for statistical analyses. The reported *p* values are two-tailed with a significance level of 5%.

## Results

### Included Data

**FIGURE 3:**
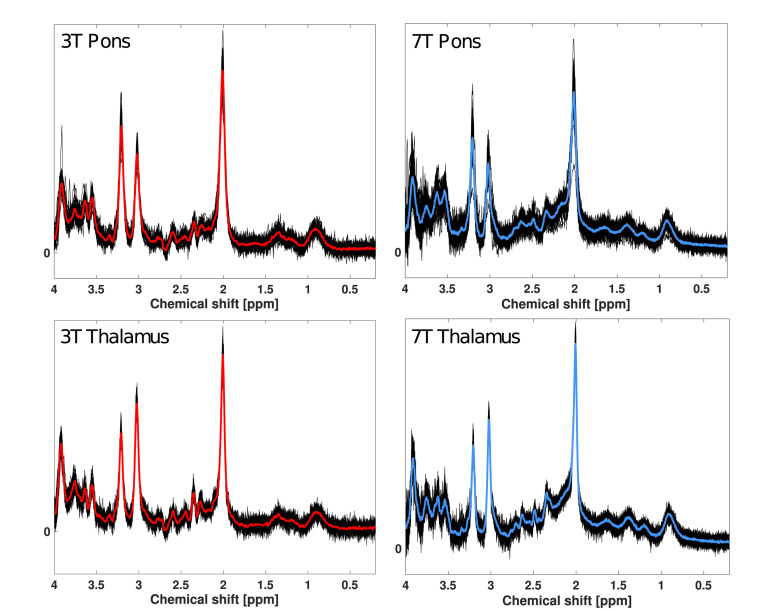
Overlay of all ^1^H-MR spectra from pons and thalamus at 3T and 7T. Black lines represent LCmodel fits of each spectrum included in analysis. The red lines represent the mean spectral fit at 3T for pons and thalamus. The blue lines represent the mean spectral fit at 7T for pons and thalamus.

The study yielded a total of 43 pontine and 45 thalamic spectra at 3T and 45 pontine and thalamic spectra at 7T for spectral quality analyses. Of these, 25 pontine and 25 thalamic spectra from both 3T and 7T were used for variability analyses. One 3T pons spectrum (scan 1) was not acquired due to technical issues, and one 3T pons spectrum (scan 1) was excluded upon visual inspection due to poor quality. Concentration estimates and corresponding CRLB were excluded for Glx and glutamine from only one 7T pons spectrum (baseline scan) as the estimate was > 3 standard deviations from the mean. All spectra are visualized in Figure 3.

### Spectral Quality

#### Pons

**FIGURE 4:**
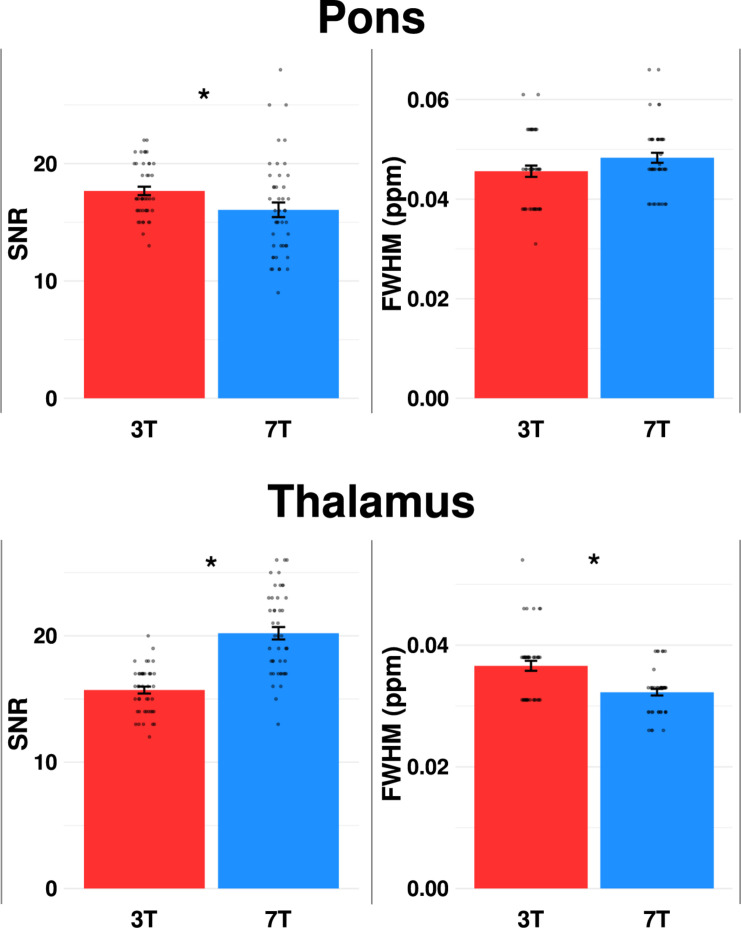
Comparison of mean signal-to-noise ratio (SNR) and linewidth [full-width of half-maximum (FWHM)] for pons and thalamus at 3 vs. 7T. Dots represent each spectral measurement. Error bars denotes standard error of the mean. *p < 0.05.

There were no differences in FWHM between 3T and 7T (*p* = 0.057), while SNR was lower at 7T (*p* = 0.006) (Table 2 and Figure 4).

**TABLE 2:** Comparison of spectral quality parameters for pons and thalamus at 3T vs. 7T.

	Pons			Thalamus		
	3T	7T	*p*	3T	7T	*p*
**SNR** (mean ± SE)	17.70 ± 0.55	16.07 ± 1.26	0.006	15.71 ± 0.51	20.20 ± 0.99	< 0.001
**FWHM** (mean ± SE)	0.05 ± 0.00	0.05 ± 0.00	0.079	0.04 ± 0.00	0.03 ± 0.00	< 0.001
**Glx**						
CRLB (mean ± SE)	11.59 ± 0.55	9.65 ± 0.61^a^	0.002	9.07 ± 0.16	5.80 ± 0.30	< 0.001
Spectra—CRLB < 20	43/43	42/44^a^	–	45/45	45/45	–
Spectra—CRLB < 50	43/43	44/44^a^	–	45/45	45/45	–
**Glutamate**						
CRLB (mean ± SE)	16.3 ± 0.61	7.89 ± 0.87	< 0.001	10.87 ± 0.22	4.78 ± 0.33	< 0.001
Spectra—CRLB < 20	34/43	45/45	–	45/45	45/45	–
Spectra—CRLB < 50	43/43	45/45	–	45/45	45/45	–
**Glutamine**						
CRLB (mean ± SE)	30.92 ± 13.81	76.01 ± 10.27^b^	0.003	26.87 ± 1.18	20.80 ± 1.61	< 0.001
Spectra—CRLB < 20	3/43	0/32^b^	–	0/45	20/45	–
Spectra—CRLB < 50	42/43	14/32^b^	–	45/45	45/45	–
**GABA**						
CRLB (mean ± SE)	88.50 ± 20.26	52.79 ± 17.60	0.090	44.89 ± 1.85	20.96 ± 1.94	< 0.001
Spectra—CRLB < 20	0/43	4/45	–	0/45	21/45	–
Spectra—CRLB < 50	5/43	35/45	–	32/45	45/45	–
**tNAA**						
CRLB (mean ± SE)	2.97 ± 0.09	1.84 ± 0.14	0.002	3.62 ± 0.10	1.89 ± 0.12	< 0.001
Spectra—CRLB < 20	43/43	45/45	–	45/45	45/45	–
Spectra—CRLB < 50	43/43	45/45	–	45/45	45/45	–
**tCreatine**						
CRLB (mean ± SE)	3.82 ± 0.12	3.38 ± 0.20	0.002	3.05 ± 0.11	2.58 ± 0.11	< 0.001
Spectra—CRLB < 20	43/43	45/45	–	45/45	45/45	–
Spectra—CRLB < 50	43/43	45/45	–	45/45	45/45	–
**tCholine**						
CRLB (mean ± SE)	3.14 ± 0.29	3.67 ± 0.22	0.076	5.07 ± 0.21	4.87 ± 0.20	0.339
Spectra—CRLB < 20	43/43	45/45	–	45/45	45/45	–
Spectra—CRLB < 50	43/43	45/45	–	45/45	45/45	–
**Myo-inositol**						
CRLB (mean ± SE)	13.05 ± 0.71	5.24 ± 0.52	< 0.001	16.87 ± 0.77	5.64 ± 0.62	< 0.001
Spectra—CRLB < 20	40/43	45/45	–	34/45	45/45	–
Spectra—CRLB < 50	43/43	45/45	–	45/45	45/45	–

*Mean CRLBs of metabolite estimates include all spectra for comparison between 3 and 7T. The numbers of spectra within the 20 as well as the 50 CRLB limits are reported for each metabolite as well. Absolute mean and standard error of the mean are reported from mixed model. ^a^One spectrum excluded due to an outlying value; two of the included spectra had a CRLB of 21. ^b^Twelve spectra excluded due to undetectable values and one due to outlying value. SE, standard error of the mean; CRLB, Cramér–Rao lower bounds; SNR, signal noise ratio; FWHM, full-width of half-maximum, ppm; Glx, glutamate + glutamine; GABA, γ-aminobutyric acid; tNAA, N-acetylaspartate + N-acetylaspartyl glutamate; tCreatine, creatine + phophocreatine; tCholine, glycerophosphocholine + phosphocholine.*

CRLB of Glx and glutamate was higher at 3T compared to 7T (*p* = 0.002 and *p* < 0.001). CRLB for glutamate was below 20 for 34 spectra at 3T compared to 45 spectra at 7T.

When applying a CRLB < 50 limit for GABA, 35 spectra at 7T were within the limit, compared to 5 spectra at 3T, yielding lower mean CRLB values at 7T (27.10 ± 1.24 vs. 42.0 ± 3.30, *p* < 0.001).

Applying CRLB < 50 limit for glutamine, we found lower CRLB at 3T (42 spectra) than 7T (14 spectra) (30.12 ± 2.93 vs. 36.52 ± 2.46, *p* = 0.040).

CRLBs of tNAA (*p* = 0.002), tCreatine (*p* = 0.002) and myo-inositol (*p* < 0.001) were lower at 7T compared to 3T. There was no difference in the CRLB of tCholine (*p* = 0.076).

Glutamine concentration estimate could not be assessed based on the low signal. Lactate signal was undetectable in the 3T and 7T spectra.

#### Thalamus

SNR was higher, while FWHM and CRLB of Glx were lower at 7T compared to 3T (*p* < 0.001). CRLB for glutamate was below 20 for all 45 spectra at 7T, while CRLB for GABA was below 50 for all 45 spectra as well at 7T.

CRLBs of tNAA, tCreatine and myo-inositol were lower at 7T compared to 3T (*p* < 0.001). There was no difference in the CRLB of tCholine (*p* = 0.339). Lactate was undetectable at 3T. Lactate at 7T was not included as it was undetectable in 56% of spectra (25 out of 45).

#### Variability of Metabolite Concentration Estimates

Five repeated measurements (free from active drugs) of the five participants per magnetic field strength were included for these analyses. As stated in methods, and confirmed by spectral quality results, concentration estimates with CRLB < 20 were reported for all metabolites, except for glutamine and GABA, where CRLB < 50 was applied.

#### Pons

**FIGURE 5:**
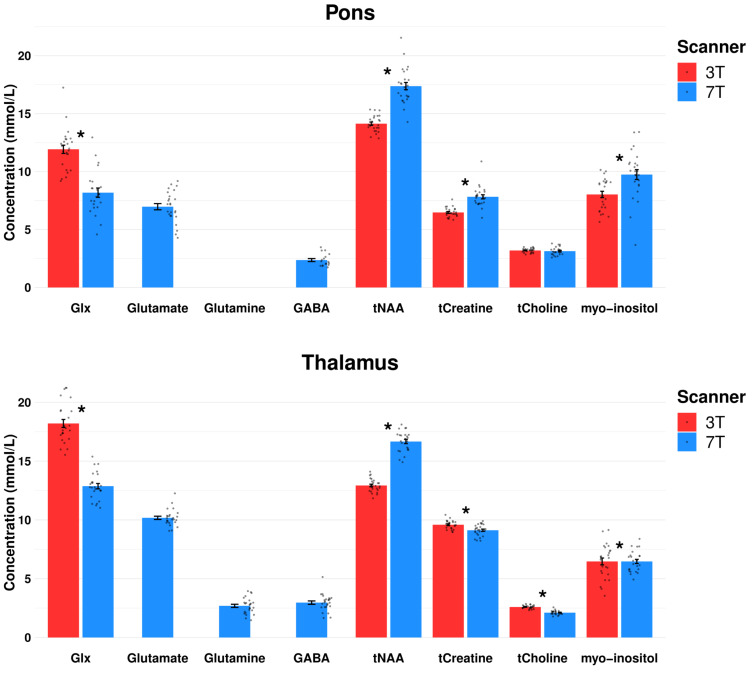
Comparison of mean metabolite concentration estimates for pons and thalamus at 3T vs. 7T of placebo day and baseline measurements. Dots represent each spectral measurement. *p < 0.05. Error bars denotes standard error of the mean, Glx, glutamate + glutamine; GABA, γ-aminobutyric acid; tNAA, *N*-acetylaspartate + *N*-acetylaspartyl glutamate; tCreatine, creatine + phophocreatine; tCholine, glycerophosphocholine + phosphocholine.

Mean within subject CV of Glx was reduced by 16% at 7T (11.7 ± 2.6) compared to 3T (14.0 ± 3.3) (Table 3). Twenty-three spectra out of 24 passed the CRLB < 20 limit for Glx at 7T. CRLB was 21 for the one 7T Glx concentration estimate, which did not pass the CRLB < 20. The pontine mean Glx levels were lower at 7T than at 3T (*p* < 0.001) (Figure 5).

**TABLE 3:** Mean within-subject coefficients of variation (%) at 3T and 7T.

	Pons				Thalamus			
	3T (mean ± SE)	Spectra (n)	7T (mean ± SE)	Spectra (n)	3T (mean ± SE)	Spectra (n)	7T (mean ± SE)	Spectra (n)
Glx	14.0 ± 3.3	25/25	11.7 ± 2.6	23/24	8.60 ± 1.49	25/25	9.1 ± 0.5	25/25
Glutamate	NA	NA	15.9 ± 3.7	25/25	NA	NA	6.5 ± 1.0	25/25
Glutamine	NA	NA	NA	NA	NA	NA	23.3 ± 3.4	25/25
GABA	NA	NA	23.5 ± 4.2	17/25	NA	NA	23.1 ± 2.2	25/25
tNAA	2.3 ± 0.5	25/25	8.1 ± 2.3	25/25	2.6 ± 0.3	25/25	2.9 ± 0.4	25/25
tCreatine	4.2 ± 0.8	25/25	9.8 ± 2.6	25/25	2.8 ± 0.3	25/25	3.6 ± 0.9	25/25
tCholine	4.5 ± 0.8	25/25	10.5 ± 1.4	25/25	4.5 ± 0.3	25/25	6.8 ± 1.2	25/25
Myo-inositol	13.0 ± 2.3	24/25	20.4 ± 3.5	25/25	17.0 ± 3.6	18/25	8.0 ± 0.6	25/25

*SE, standard error of the mean; Glx, glutamate + glutamine; GABA, γ-aminobutyric acid; tNAA, N-acetylaspartate + N-acetylaspartyl glutamate; tCreatine, creatine + phophocreatine; tCholine, glycerophosphocholine + phosphocholine; n, numbers; NA, not applicable.*

#### Thalamus

Mean within-subject CV of Glx at 7T was reduced by 17% compared to 3T. All concentration estimates passed their CRLB-limits (25 out of 25 spectra for 3T and 7T). Mean Glx concentration estimates were lower at 7T compared to 3T in thalamus (*p* = 0.011).

## Discussion

The major findings of the present study were successful separation of glutamate from glutamine and detection of GABA at 7T in the deep, subcortical structures pons and thalamus. Furthermore, spectral quality was improved at 7T in thalamus, as compared to 3T, while in pons, a similar quality was achieved with a total scan time of ∼12 min compared to ∼34 min.

### Spectral Quality Assessment

Detection of glutamate and GABA non-invasively and *in vivo* in humans is of great interest in several brain disorders, including neurological as well as psychological entities (Foerster et al., 2013; Brennan et al., 2017; Prisciandaro et al., 2017; Younis et al., 2018). Changes in glutamate and/or GABA within pons and thalamus are considered important in the underlying pathophysiology of several of these various brain disorders (Pioro et al., 1999; Foerster et al., 2013; Adanyeguh et al., 2015; Neill et al., 2016; Bathel et al., 2018; Younis et al., 2018). Since the combination of obtaining weak signal molecule from deep brain regions is technically arduous, and consequently time-consuming, researchers may hold a tendency toward avoiding investigations of glutamate and GABA within these structures. This is despite the clinical relevance with potential pathological revelations and possibly development of new treatment options by carrying out the investigations.

The spectral quality was assessed based on CRLB, SNR as well as the spectral linewidth (FWHM) between 3T and 7T spectra. Here, we showed that glutamate and GABA were detectable at 7T in pons and thalamus with a feasible total scan time of 12 min. CRLB levels were below the expected levels for glutamate in 100% of pontine and thalamic spectra at 7T and 78%–100% for GABA. Mean CRLBs of glutamate in pons and thalamus were 7.9 and 4.8, while CRLBs were 27 and 21 for GABA in respective regions. For comparison, one 7T study reported CRLB of ∼2.5 for glutamate and ∼15 for GABA in cerebellum (Terpstra et al., 2016), another challenging area to measure with ^1^H-MRS, while two other studies reported CRLB of ∼3 for glutamate and ∼7–29 for GABA in posterior cingulate cortex (van de Bank et al., 2015; Wijtenburg et al., 2019) and, by sLASER, ∼4 for glutamate in dorsal anterior cingulate cortex (Dempster et al., 2020). Compared to these less technically challenging ^1^H-MRS regions, our CRLBs for pontine and thalamic glutamate and GABA investigations were well-estimated at 7T. Additionally, the glutamate and GABA levels were detectable within the same spectrum, allowing assessment of dynamics between the excitatory and inhibitory mechanisms simultaneously. This further decreases the total scan time in cases where researchers are interested in measuring both metabolites.

The CRLB was lower at 7T for all detectable metabolites, except total choline, in both pons and thalamus, suggesting an overall improved spectral fit in pons and thalamus (Tkác et al., 2001; Tkáč et al., 2009; Deelchand et al., 2014; Terpstra et al., 2016; Provencher, 2018). This further suggests an overall improved spectral quality at 7T in pons and thalamus.

Previous studies comparing spectral quality between cortical regions at 3T and 7T likewise report improved spectral outputs with increasing field strengths and consequently improved quantification of glutamate (Mekle et al., 2009; Pradhan et al., 2015; Terpstra et al., 2016). Here, we confirm that this is also applicable for higher field ^1^H-MRS in pons and thalamus, despite the well-known increased risk of susceptibility effects in those areas at 7T. Interestingly, glutamine also appeared to be detectable at 7T in thalamus, based on the average CRLB and high number of spectra below the CRLB limit of 50. On the other hand, the CRLB of pontine glutamine was higher at 7T than 3T. The higher CRLB of glutamine at 7T may indicate that assessment of glutamine at 3T is biased by spectral overlap with, e.g., glutamate. Thus, it might be that pontine glutamine is being overestimated at 3T leading to lower CRLB. The quality of the glutamate fit was better at 7T and may have resulted in a more accurate glutamine fit. Moreover, the mean CRLB of glutamate at 3T was below 20 with > 50% of spectra passing the CRLB limit of 20. While this LCModel fit implies that glutamate is detectable at 3T, this is most likely not the case since glutamate and glutamine is not reliably separated at magnetic field strengths < 4T (Deelchand et al., 2014). Therefore, we deemed that both glutamate and glutamine were unreliable at 3T, while glutamine was unreliable at 7T in the pons, based on our acquisition protocols. In our study, we achieved a 42% gain in SNR and 11% reduction in FWHM at 7T in thalamus, demonstrating improved spectral quality at 7T vs. 3T. This was obtained by applying three times fewer acquisitions at 7T as compared to 3T, meaning a total 2.25-fold gain in SNR/sqrt (time). For comparison, a previous ^1^H-MRS study reported only a 22% SNR gain in cerebellum at 7T compared to 3T, another area that is challenging to measure with ^1^H-MRS (Terpstra et al., 2016). For the pontine spectral measurements, no gain in SNR or decrease in FWHM was observed. The spectra were obtained by 3.75 times fewer acquisitions, meaning a total 1.76-fold gain in SNR/sqrt(time). This lower SNR gain in pons, compared to thalamus, may be attributed to susceptibility effects from the nearby air cavities (Sclocco et al., 2018). Increased chemical shift dispersion at higher field strengths (Tkác et al., 2001, Tkáč et al., 2009; Deelchand et al., 2014; Terpstra et al., 2016) may explain why detection of signal from weaker metabolites, such as the glutamate and GABA resonances in pons, are possible at 7T, despite SNR and FWHM not being significantly better than the spectra acquired at 3T. Moreover, SNR and FWHM are only rough estimators of spectral quality, while CRLB accounts for the spectral noise levels as well as the resolution, providing a favorable assessor for metabolic quantification precision (Provencher, 2018). Furthermore, intrinsic SNR may increase 1.7-fold from 3T to 7T (Mekle et al., 2009), and reach a nearly constant level above 3–4T (Deelchand et al., 2014), which may explain the absent SNR increase in pons. However, gain in quantification precision seems to continuously increase with higher field strengths (Deelchand et al., 2014), as reflected in the overall improved CRLB levels at 7T compared to 3T.

### Glutamate and GABA Estimations

While demonstrating that glutamate and GABA are detectable at 7T in the pontine and thalamic spectra, it is also important to assess the variability of the measurements before introducing the method to future human studies of pontine and thalamic glutamate and GABA investigations. This is especially relevant in longitudinal study designs with repeated measurements, e.g., studies with efficacy assessment of pharmacological treatment or other interventions. For this reason, we estimated the mean within-subject CV for glutamate and GABA.

We showed that mean within-subject CV of glutamate in pons was 15.9%, while 6.5% in thalamus. This difference is expected as pons is a more subcortical brain region than thalamus. Since glutamate and GABA were undetectable at 3T, we evaluated the CV of Glx between the two field strengths. We found a 1.2 times lower mean within-subject CV for Glx at 7T compared to 3T in pons and no difference in thalamus.

A limited number of studies investigating the variability of glutamate at 7T are available for comparison (Cai et al., 2012; Terpstra et al., 2016; Wijtenburg et al., 2019; Dempster et al., 2020). In addition, these studies apply larger MRS voxels with more cortical placement than in the current study. One 3T vs. 7T ^1^H-MRS study also suggested lower test–retest CV (∼5% vs. ∼4%) of cerebellar glutamate at 7T compared to 3T when using 64 acquisitions with sLASER (Terpstra et al., 2016). Another 7T study, in the more superficial visual cortex, measured variability in glutamate levels between two repeated measurements on the same day (∼12%) and 2 weeks apart (∼11%), which were comparable to our findings (Cai et al., 2012). This study used PRESS with 100 acquisitions for the glutamate measurements (Cai et al., 2012). A more recent 7T study of anterior and posterior cingulate cortex reported CV of 6–8%, however, the results were reported as relative estimate to total creatine by STEAM and 96 acquisitions (Wijtenburg et al., 2019). Another 7T study reported within-subject glutamate variation of 20% measured in dorsal anterior cingulate cortex measured 1 month apart using sLASER with 32 acquisitions (Dempster et al., 2020).

*Post hoc* power analysis, based on the CVs of glutamate found in our study, showed that with a sample size of 25, it would be possible to detect a 3.8% change in glutamate levels in thalamus and 9.3% in pons. This example demonstrates that applying ^1^H-MRS in pons and thalamus is promising in future clinical research using repeated measurement designs.

Variability of GABA measurements was expectedly higher in our study (23.1%–23.5%) than glutamate due to the weaker GABA signal. Similar to the glutamate variability assessment, few studies are available for comparison, while measurements are performed from more cortical structures and larger MRS voxels as well (Cai et al., 2012; Terpstra et al., 2016; Li et al., 2017; Wijtenburg et al., 2019).

One 7T study reported comparable test–retest CV for GABA of ∼17%–22% (estimated from figure) when measured in posterior cingulate cortex and cerebellum (Terpstra et al., 2016). Another 7T study of anterior and posterior cingulate cortex reported CV of ∼18% (Wijtenburg et al., 2019). These findings were reported as relative estimate to total creatine by STEAM and 96 acquisitions (Wijtenburg et al., 2019). Variability of GABA measured in visual cortex was ∼5% for two repeated measurements on the same day and ∼6% for measurement 2 weeks apart (Cai et al., 2012). This study used MEGA-PRESS with 100 acquisitions and found lower CVs, most likely due to location in visual cortex and larger MRS voxel (Cai et al., 2012). Another 7T also used edited PRESS sequence and found a variability of GABA at ∼17% in caudate (Li et al., 2017), comparable to our findings. One 7T ^1^H-MRS multi-center study of two repeated measurements in corona radiata and posterior cingulate cortex, reported an 18%–22% test–retest CV for GABA and ∼3% for glutamate (van de Bank et al., 2015). This study also implied higher GABA variability compared to glutamate, despite measurement in brain areas less prone to susceptibility effects.

When applying CV of GABA from our study in *post hoc* analysis, using sample size of 25, we demonstrate the possibility of detecting at least a 13.7% change of GABA in thalamus and 13.5% in pons, which could prove sufficient for many studies (Cai et al., 2012).

We also demonstrate that glutamate and GABA levels can be obtained simultaneously using a non-edited ^1^H-MRS sequence in the thalamus as well as in the pons. GABA measurements by edited sequences are typically more challenging compared to non-edited sequences (Puts and Edden, 2012), i.e. GABA-edited sequences are more sensitive to motion instabilities due to subtraction of the signals (Puts and Edden, 2012). One longitudinal 7T study of two repeated measurements found lower CV for GABA in anterior cingulate cortex (3.5 vs. 13.6%), but higher CV for GABA in dorsolateral prefrontal cortex (16.2 vs. 13.4%) using a non–GABA-edited sequence, as opposed to a GABA-edited sequence (Wijtenburg et al., 2013).

### Other Metabolites

Detection of other metabolites was generally improved at 7T in both pons and thalamus (Table 2). Another interesting aspect is, however, that the CVs of the more abundant signals, such as total creatine and total NAA, were slightly higher at 7T than at 3T. Similar difference in concentration estimates has previously been reported in a test–retest study of ^1^H-MRS in cerebellum and posterior cingulate cortex between 3T and 7T (Terpstra et al., 2016). We speculate that this may be due to a more precise estimate of the concentrations at 7T leading to the increased variability between the within-subject measurements. On the other hand, we may also consider the 10-month span between the 3T and 7T data collection, which might influence the difference in variability estimates as well as the absolute concentrations estimates visualized in Figure 5.

### Considerations

Here we report a novel study comparing the feasibility and quality of ^1^H-MRS measurement of pontine and thalamic glutamate and GABA at 3T and 7T with a longitudinal design, which to our knowledge, have not been previously reported. We showed that GABA and glutamate can be estimated using non-edited ^1^H-MRS at 7T using 3- to 4-fold shorter scan times than at 3T, which is beneficial for pharmacokinetic investigations of brain excitatory–inhibitory mechanisms in a clinical setting.

Optimal measurement of low signal metabolites in deep brain structures, such as pons and thalamus, relies on superior technical efforts due to regionally increased magnetic field inhomogeneity, near the cranial base, and due to physiological artifacts. ^1^H-MR spectral quality in these regions is, therefore, commonly inferior to cortical brain regions (Terpstra et al., 2016) and requires a higher number of acquisitions, which prolongs scan times. Thus, 3T vs. 7T ^1^H-MRS comparison studies of cortical structures are not readily applicable to pontine and thalamic measurements. In the present study, we applied widely used and available ^1^H-MRS sequences tailored for use at 3T and 7T in the pons and thalamus. Scan times at 7T were reduced 3.75-fold for pons and 3-fold for thalamus compared to 3T to ameliorate the total scan durations in future clinical settings.

TR may play a subtle role in SNR. As far as we know, there is no knowledge about T_1_ relaxation time differences in the pons and thalamus to perform TR corrections. We used comparable TR’s at both field strengths, however, due to the SAR limitations, TR at 7T was increased by 20%. Nevertheless, we expect that the impact of the increased TR at 7T on the SNR is minimal as the T_1_ relaxation times are also expected to be increased. In addition, optimal sequences and shimming procedures were used at both field strengths. We used PRESS sequence at 3T to opt for a clinically feasible approach. sLASER and SPECIAL may be used to estimate glutamate levels at 3T (Öz et al., 2020). However, these sequences are not standard on clinical 3T scanners and therefore not readily applicable. We did not correct for tissue type, since the voxels were placed in the homogenous brain tissue regions of pons and thalamus. Moreover, we were investigating within-subject measurements between field strengths. SNR was obtained from LCModel. To minimize patient motion, pads were applied within the head-coil to restrict movement, while it was ensured that patients were comfortable as possible before initiating the scans. Moreover, patients were carefully instructed to remain still during the scans.

Another question is whether reduced scan time might impact the spectral quality. Exploring this is, however, unattainable as glutamate levels, as well as GABA levels using non-edited sequences, are very challenging to reliably estimate at 3T.

## Conclusion

Glutamate can be reliably measured in pons and thalamus at 7T. Spectral quality and variability of pontine and thalamic ^1^H-MRS was overall improved at 7T compared to 3T with a 3- to 4-fold shorter scan time. In addition, we demonstrated that measurement of GABA, simultaneously with glutamate, in pons and thalamus is feasible using non-edited ^1^H-MRS for pharmacokinetic investigations of brain excitatory–inhibitory mechanisms. 7T provides the opportunity to improve spectral quality even more by extending scan durations. Furthermore, the shorter scan times at 7T open up the opportunity to acquire measurements from multiple brain regions.

## Data Availability Statement

All datasets presented in this study are included in the article and is available upon reasonable request to the corresponding authors.

## Ethics Statement

The study was reviewed and approved by the Danish National Committee on Health Research Ethics (H-15019063) and the Danish Medicines Agency (CIV-16-12-017964). The participants provided their written informed consent to participate in this study.

## Author Contributions

SY, AH, CEC, MBV, ETP, VOB, OBP, MA, AM, and HBWL: conceptualization. SY and CEC: data curation. SY, MBV, and AM: formal analysis. AH, HBWL, and MA: supervision. SY: writing – original draft. SY, AH, MBV, ETP, VOB, OBP, MA, AM, and HBWL: writing – review and editing. All authors contributed to the article and approved the submitted version.

## Conflict of Interest

The authors declare that the research was conducted in the absence of any commercial or financial relationships that could be construed as a potential conflict of interest. The reviewer MW declared a past collaboration with one of the authors VOB to the handling editor.

## Footnotes

1 www.forsoegsperson.dk

## Abbreviations

^1^H-MRS: proton magnetic resonance spectroscopy
CGRP: calcitonin gene-related peptide
CRLB: Cramér–Rao lower bounds
CV: coefficient of variation
FWHM: full-width of half-maximum
GABA: γ-aminobutyric acid
Gln: glutamine
Glu: glutamate
Glx: glutamate + glutamine
*ICHD-3* beta: diagnostic criteria of the beta version of the third *International Classification of Headache Disorders*
mIns: myo-inositol
PRESS: point-resolved spectroscopy
SAR: specific absorption rate
SE: standard error of the mean
sLASER: semilocalization by adiabatic-selective refocusing
SNR: signal-to-noise ratio
tCholine: glycerophosphocholine + phosphocholine
tCreatine: creatine + phosphocreatine
tNAA: *N-*acetylaspartate + *N*-acetylaspartylglutamate.
